# Generalization of Human Fear Acquisition and Extinction within a Novel Arbitrary Stimulus Category

**DOI:** 10.1371/journal.pone.0096569

**Published:** 2014-05-05

**Authors:** Ellen Vervoort, Bram Vervliet, Marc Bennett, Frank Baeyens

**Affiliations:** Centre for the Psychology of Learning and Experimental Psychopathology, Faculty of Psychology and Educational Sciences, University of Leuven, Leuven, Belgium; Swansea University, United Kingdom

## Abstract

Adaptive anxiety relies on a balance between the generalization of fear acquisition and fear extinction. Research on fear (extinction) generalization has focused mostly on perceptual similarity, thereby ignoring the importance of conceptual stimulus relations in humans. The present study used a laboratory procedure to create de novo conceptual categories of arbitrary stimuli and investigated fear and extinction generalization among these stimuli. A matching-to-sample task produced two four-member categories of abstract figures. Next, a member from one category was coupled with an aversive electrical stimulation, while a member from the other category was presented alone. As expected, conditioned fear responses generalized to the other members of the first category (skin conductance and online shock-expectancy). Subsequent extinction of the conditioned member also generalized to the other members. However, extinguishing a non-conditioned member failed to reduce fear of the conditioned member itself. We conclude that fears generalize readily across conceptually related stimuli, but that the degree of extinction generalization depends on the stimulus subjected to extinction.

## Introduction

Traumatic experiences can result in fearful reactions to a wide range of trauma-related stimuli, even if they are themselves innocuous [1]. This is modeled in the Pavlovian fear conditioning procedure where pairings of a neutral stimulus (conditioned stimulus, CS) and an aversive stimulus (unconditioned stimulus, US) result in the subsequent expression of fear towards the CS. The CS and US have arguably become associated in memory, thereby establishing the CS as a predictive signal of the aversive US and eliciting fear of that specific CS. In many cases, however, traumatic experiences will yield fears of stimuli far beyond the actual traumatic situation [2]. This generalization of fear can greatly increase the impact of a traumatic event and pose a great burden on daily life [3], . It may also complicate psychotherapeutic interventions. Extinction-based psychotherapies involve repeated exposures to fear-arousing situations until the fear declines [5]. This technique is highly efficacious but the question is to what extent it remedies the entire range of generalized fear-eliciting situations and events. Previous research has shown that while conditioned fears generalize easily over perceptually related stimuli, extinction of fear does not [6], [7]. The present study focuses on *conceptual* relations between stimuli as a source of fear generalization and investigates the general impact of extinction with a target stimulus.

Generalization occurs when a conditioned (fear) response is elicited by a stimulus different from the actual CS [8]. For instance, stimuli that bear physical similarity with the CS will typically evoke a certain degree of conditioned responding (e.g., [3], [6]–[7], [9]). As well as this perceptual-based interaction, humans have a tendency to approach stimuli in a conceptual way. Dunsmoor, White, and LaBar [10] found that conceptual similarities between stimuli (e.g., spider and web) enhanced the generalization of conditioned fear. That is, learned fear generalized more easily between these stimuli compared with unrelated stimuli. The ability to link stimuli according to conceptual knowledge plays an important role in the acquisition and generalization of new learning and it constitutes a significant extension of the generalization research field. However, the use of naturalistic concepts and categories has the disadvantage of diminished experimental control over their learning histories [10]. Often, members of one category will have been experienced together (e.g., spider-web), leaving the possibility open that the generalization follows directly experienced associations rather than abstract category membership. We decided to investigate fear generalization with de novo created stimulus categories, in order to maximize experimental control. This excludes any influence of previous pairings of these stimuli. We also controlled for perceptual overlap between stimuli, by selecting entirely arbitrary stimuli. In addition, the current study broadened the focus to include generalization of fear extinction. This allows for the balance between acquisition and extinction generalization among members of stimulus categories to be studied.

For the above purposes, we combined the standard Pavlovian fear conditioning procedure with a version of the matching-to-sample (MTS) task (e.g., [11], [12]). This is an operant conditioning procedure through which a number of arbitrary *‘comparison’* stimuli are directly related to one central *‘sample’* stimulus. Following this training, stimulus relations that had never been explicitly trained can emerge spontaneously. These derived relations entail a reversal of the trained relation such that samples are related to comparisons (known as *symmetry*) and a combination of the trained relations such that comparisons become related to one other (known as *equivalence*) [11]. As these stimuli become functionally substitutable a new category is said to be formed [13]. Previous research on stimulus equivalence has demonstrated that new behaviors trained to one member of a category will generalize to the other members without ever having been experienced together and without any perceptual overlap (e.g., [14]–[20]).

An earlier study by Dougher, Augustson, Markham, Greenway, and Wulfert [16] demonstrated generalization of both conditioned fear and extinction among members of the same de novo created category. However, the conclusions were based on a very small number of participants (N = 8) and only at face value (no statistical tests). This contrasts with contemporary research on perceptual generalization of fear (extinction) that uses larger numbers of participants and adequate statistical testing methods. Valverde, Luciano, and Barnes-Holmes [21] have replicated the generalization of acquisition effect found by Dougher et al., using conditioning parameters and statistical analyses consistent with contemporary research standards. However, given the potential importance of this phenomenon in the development of clinical anxiety, more experimental evidence is desirable. In addition, Dougher et al. investigated generalization of extinction after conditioning all members of one and the same category. Extinction generalization is typically investigated by extinguishing only one generalization stimulus followed by tests of the original CS and other generalization stimuli [6], [7], . For instance, Roche, Kanter, Brown, Dymond, and Fogarty [25] extinguished either the original CS or one generalization stimulus and then examined the generalization of extinction to related members. However, significant procedural differences between this study and the study by Dougher et al. make it difficult to deduce unambiguous conclusions about extinction generalization. The current research method constitutes a combination of these two studies and can therefore shed light on this matter.

The current study used the procedure of Dougher et al. ([16], Experiment 1) to investigate category-based generalization of fear and extinction with contemporary standards derived from perceptual generalization research. An electrical stimulus served as aversive US. Fear reactions were measured implicitly through skin conductance responses and explicitly through trial-by-trial shock expectancy ratings. We hypothesized that stimuli conceptually related to a stimulus paired with the US (referred to as CS+) would elicit higher skin conductance responses and US expectancy ratings than stimuli related to a stimulus that is never followed by the US (referred to as CS-). It was further assessed to what extent extinction generalizes to the other stimuli of a category. More specifically, extinction with the original CS+ was compared to extinction with a generalization stimulus.

## Methods and Materials

**Figure 1:**
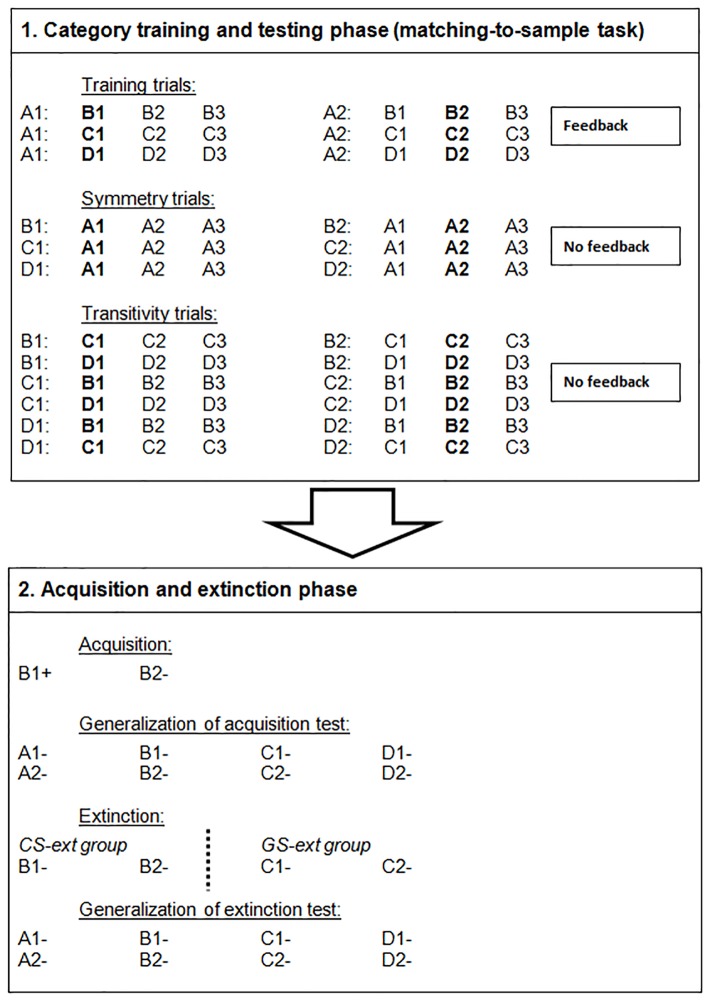
A schematic overview of the experimental phases. The upper panel represents the trials used in the matching-to-sample training, symmetry test and equivalence test, in order to create two novel stimulus categories (A1-B1-C1-D1 and A2-B2-C2-D2). The first stimulus always represents the sample stimulus, the other three stimuli are the comparison stimuli. The correct comparison stimulus is indicated in bold. The lower panel represents the fear conditioning phase (acquisition, generalization of acquisition test, extinction, generalization of extinction test). The “+”sign indicates that this stimulus is followed by an electric shock in 8 out of 10 trials. The “−”sign indicates that this stimulus is never followed by a shock during those trials.

The study consisted of two phases (See Figure 1). In the first phase two four-member stimulus categories were established using a MTS procedure. The second phase involved a classical conditioning procedure. It consisted of an acquisition phase and an extinction phase, involving stimuli from both categories. Both after acquisition and after extinction, all stimuli were presented once, to test for generalization of the conditioned responses through implicit stimulus relations derived during the MTS task.

### Ethics statement

This study was approved by the local ethics committee of the University of Leuven (Faculty of Psychology and Educational Sciences). All participants signed a written informed consent. The minimum age to participate was set at 18.

### Participants

Fifty-one psychology students (39 females) participated in the experiment. They could choose to be reimbursed with course credits or 8 euro/hour. Sixteen of these participants were excluded during the experiment as they did not meet the pre-established criteria in the first phases (see below). The 35 participants (27 females) who did succeed were randomly assigned to two experimental groups: GS-ext group (N = 18, mean age  = 19.611, SD = 2.19) and CS-ext group (N = 17, mean age = 19.941, SD = 3.523).

### Apparatus

**Figure 2:**
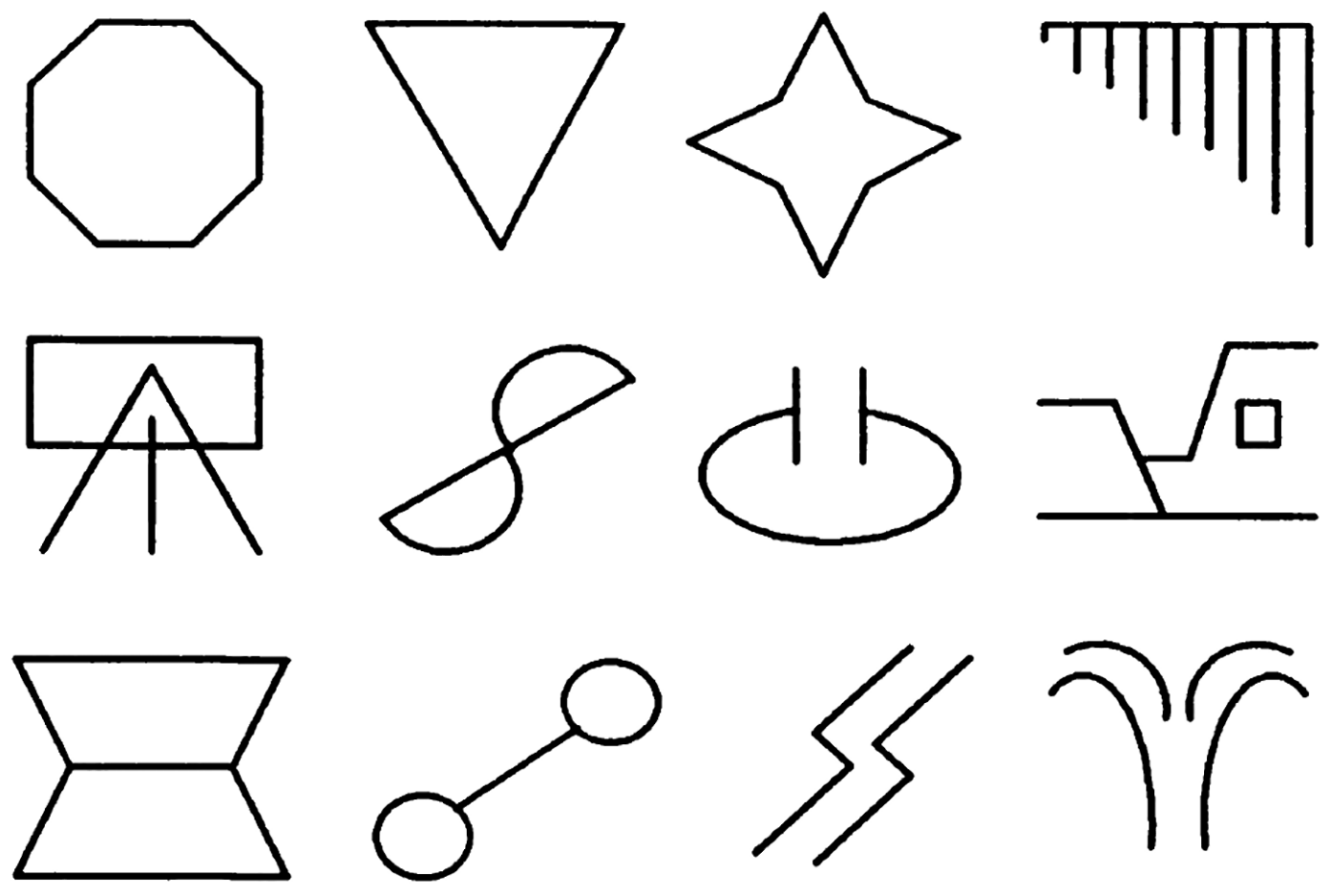
Abstract figures used in the experiment to create novel categories.

The stimuli used in this experiment were 12 abstract geometrical figures (See Figure 2). Eight of these stimuli were divided into two arbitrary categories each containing four figures. The selection of these eight stimuli and the composition of the categories were randomized. All stimuli will be represented alphanumerically based on the category (category 1 = CAT+ = A1, B1, C1, D1, category 2 = CAT- = A2, B2, C2, D2 and four remaining stimuli  = A3, B3, C3, D3). All stimuli, 4×4 cm, were black on a white background and were presented on a computer screen. The screen was located at eye-level and the distance from the participant was approximately 50 cm. Presentation of the stimuli was controlled by Affect3 software [26]. A 2 ms electro-cutaneous stimulus served as unconditioned stimulus (US). It was delivered by a Digitimer DS7A constant current stimulator (Hertfordshire, UK) via a pair of 11-mm Fukuda Standard Ag/AgCl electrodes to the wrist of the left hand. The electrodes were filled with K-Y jelly. The intensity of the shock was determined by the participant as “uncomfortable, but not painful” (*M* intensity  = 1.63 mA; *SD*  = .99). A skin conductance coupler from Coulbourn Instruments (model V71-23, Allentown, PA) was used to record electrodermal activity during the experiment. While measuring this skin conductance, the coupler applied a constant voltage of 0.5 V across a pair of sintered-pellet silver chloride electrodes (8 mm), with a distance of approximately 7 mm between them. These were attached to the palm of the left hand, which was first cleaned with tap water. These electrodes were also filled with K–Y jelly. The resulting conductance signal was submitted through a Labmaster DMA 12-bit analog-to-digital converter (Scientific Solutions, Solon, Ohio) and digitized at 10 HZ from 2 s prior to CS onset until 8 s after CS offset. Participants used their right hand to operate the computer mouse in order to indicate expectancy ratings. This was done via an 11-point scale which appeared on the bottom of the computer screen; 0 (unlikely) to 10 (very likely).

### Procedure

After completion of the informed consent participants were led to the experimental room. Electrodes were then fitted and a work-up procedure was used to select a shock intensity that was “definitely uncomfortable but not painful”. Next, participants were informed that no shocks would occur in the first task of the experiment, and that when this task was done, new instructions would appear which would warn them of the possibility of electrical stimulation. After this information, the experiment itself began with on-screen Dutch instructions:

*“When the experiment begins, you will see sets of four symbols on the screen; one at the top and three at the bottom – one on the left, one in the middle, and one on the right. Your task is to choose the correct symbol at the bottom of the screen by pressing the numeric keys 1, 2 or 3. During the first part of this phase you will get feedback on every choice. Later you will not get feedback every time. However, there is always a correct answer. During the first part of this phase the task will be easy and it is tempting not to pay attention. However, the experiment will increase in difficulty, and choosing the correct symbols in the latter part of this phase will depend on the knowledge you gain during the early parts of the experiment. Things that you learn in this part of the study may be important later on.”*

#### Category training and testing phase (matching-to-sample task)

The first phase was divided into three parts (see Figure 1). The aim of this phase was to create two four-member stimulus equivalence categories. All trials in this phase began with a *sample stimulus* appearing on the top of the screen. After 2 s, 3 *comparison stimuli* appeared in a row on the bottom of the screen whose position from left to right was randomized. Participants could choose a comparison stimulus by pressing the corresponding numeric key; key 1 for the stimulus on the left, key 2 for the stimulus in the middle and key 3 for the stimulus on the right. A key press removed all stimuli and the screen turned black. During training trials feedback would then occur (“Correct” or “Wrong”) for 5 s followed by a 2 second inter-trial interval (ITI). During testing trials no feedback would follow a response.

The MTS task started with a *training phase* which taught participants six stimulus relations (A1-B1, A1-C1, A1-D1, A2-B2, A2-C2, A2-D2). These trials were introduced in blocks, each containing 6 different trial types in random order: A1-**B1**/B2/B3, A1-**C1**/C2/C3, A1-**D1**/D2/D3, A2-B1/**B2**/B3, A2-C1/**C2**/C3, A2-D1/**D2**/D3. Note that the stimuli in bold are the correct comparison stimuli in presence of the accompanying sample stimulus. Stimuli B3, C3 and D3 were only used as third, incorrect, comparisons to reduce the likelihood that participants could learn the correct responses by exclusion. Stimulus A3 had the same function in the next parts of the MTS phase. These stimuli played no further role in the fear conditioning part of the experiment. To pass the initial training phase, 46 trials had to be correct in a consecutive series of 48 trials. If this was the case, then the participant moved on to the second part of the category phase that consisted of *symmetry* test trials. Here, A1, A2 and A3 served as comparison stimuli, while B1, C1, D1, B2, C2 and D2 now served as sample stimuli. The relations tested for in this phase were B1-A1, C1-A1, D1-A1, B2-A2, C2-A2, D2-A2. There were six trials in a single block presented randomly : B1-**A1**/A2/A3, C1-**A1**/A2/A3, D1-**A1**/A2/A3, B2-A1/**A2**/A3, C2-A1/**A2**/A3, D2-A1/**A2**/A3. As aforementioned, feedback was no longer given following testing trials. Participants had to achieve 16 correct trials out of 18 to move on to the next test phase. This part consisted of blocks of 18 trial types. These included the 6 symmetry trial types from the previous phase and 12 new *equivalence* trial types. Equivalence trials tested for the emergence of relations between the B-, C- and D-stimuli: B1-**C1**/C2/C3, B1-**D1**/D2/D3, C1-**B1**/B2/B3, C1-**D1**/D2/D3, D1-**B1**/B2/B3, D1-**C1**/C2/C3, B2-C1/**C2**/C3, B2-D1/**D2**/D3, C2-B1/**B2**/B3, C2-D1/**D2**/D3, D2-B1/**B2**/B3, D2-C1/**C2**/C3. The next phase began once in at least 34 out of 36 trials correct responses were made. The criteria used in the category training and testing phases were based upon pilot work, in which all participants meeting these criteria appeared to be able to reconstruct the categories afterwards. All participants in this pilot study who succeeded on this task, managed to do this within 30 minutes. Therefore, participants had a time limit of 30 minutes in the experiment itself to complete this phase. If they ran out of time, or in other words, if they did not meet the criteria during training or test, they were excluded from the rest of the experiment.

#### Acquisition and extinction phase

New instructions initiated the acquisition phase:

*“The first phase of the experiment has been successfully completed! Now we are moving on to the second phase. From time to time, you will see a figure on the screen. Some figures will sometimes be followed by an electric shock, other figures will not. It is your task to report whether you expect a shock after these stimuli or not, by indicating a certain spot on a scale from 0 to 100, which will appear on the bottom of the screen. You can use your free hand to do this. For every figure, you have a couple of seconds to give an answer.”*

During this phase, stimuli B1 and B2 were each presented 10 times in random order. Each stimulus was presented for 8 s followed by an ITI randomized between 10 and 15 s. Eight out of 10 B1 presentations were immediately followed by a shock (US) while 2 out of 10 were not. B1 was therefore aversively conditioned to predict US. One of the two B1 conditioning trials without a shock always took place in the first half of this phase, while the other one occurred in the second half. This 80% contingency between B1 and shock was applied to attenuate extinction learning during the subsequent generalization test. B2 was never followed by the shock.

During the presentation of a stimulus, the expectancy scale appeared on the bottom of the screen. In this 8 second period, the participants had to indicate the likelihood of a US onset on a scale between 0 and 100. The left extreme on the scale (number 0) was labeled “Certainly no shock” while the right extreme (number 100), was labeled “Certainly a shock”. The intermediate point of the scale (number 50) was labeled “Uncertain”. Participants could click on a certain spot on the scale to indicate a value. After doing this, a dot would appear on that spot, which could still be replaced if the participant changed his or her mind. Each time a new trial began the dot was removed and had to be placed on the scale again. This trial pattern, the stimulus durations and the inter trial interval were the same throughout the rest of the experiment.

Stimuli from CAT+ (A1, B1, C1 and D1) and CAT- (A2, B2, C2 and D2) were then presented once each in the absence of a US in order to test for generalization of conditioned fear. They appeared quasi-randomly onscreen for 8 s followed by a 10–15 s ITI. Participants were first exposed to the B and C stimuli. These were presented in two possible patterns: B2-B1-C2-C1 or C2-C1-B2-B1. A stimulus from CAT- was always presented first, to reduce the effects of extinction during generalization testing. After presenting these 4 stimuli, the other stimuli (A1, A2, D1 and D2) were presented in random order.

One group was subsequently presented with B1 and B2 in extinction (CS-ext Group). A second group was presented with two related stimuli, C1 and C2, also in extinction (GS-ext group). Each stimulus was presented 15 times and all trials were randomized. The US never followed a stimulus.

Finally, a second generalization test took place. Again, all stimuli from both categories were presented once, without being followed by a shock. The sequence of these trials was determined by the same rules as in the test phase after acquisition.

Immediately after the experiment, a manipulation check took place to verify whether the participants could reconstruct the two stimulus categories they had learned during the MTS task. Participants were handed 12 flash cards with the abstract figures (A1, B1, C1, A2, B2, C3, A3, B3, C4) printed on each. Their task was to divide them on a table into their constituent categories. No further information was given about the number or the size of the groups.

### Data analysis

The US expectancy ratings were registered at the moment the stimulus and expectancy scale disappeared from the screen. For the skin conductance response, amplitudes were measured as the peak value in every trial within the 0–7.5 s interval after CS onset relative to a baseline averaged over the 2 s prior to CS onset. Negative values were converted into zero and were also included in the analyses. These amplitudes were then range corrected using the largest response elicited by the US, in the 9–14 s interval after CS onset as the maximum range for each participant. Every amplitude was divided by this maximum US response. Prior to statistical analysis, the obtained values were normalized, using a square root transformation. The alpha-level was set at .05 for all analyses. Where Mauchly's test revealed that sphericity could not be assumed the Greenhouse-Geisser correction is reported. When two or more significant comparisons are described, only the values of the least significant comparison will be reported. Analogical to this, when two or more non-significant comparisons are described, only the values of the comparison that approached significance the most are reported.

## Results

All the participants who succeeded in the MTS training and testing, also did so in the manipulation check at the end of the experiment. The skin conductance responses of two participants were excluded from the analysis because of technical difficulties.

### Category training and testing phase (matching-to-sample task)

No differences were found between the conditions in the mean number of matching-to-sample training and test trials, *t*(34) <1.00, *p*>.32, as was expected (CS-ext group: mean number of training trials  = 96, SD = 30.00; symmetry test trials  = 27, SD = 26.38; equivalence test trials  = 45, SD  = 21.93; GS-ext group: mean number of training trials  = 93, SD  = 32.03; symmetry test trials  = 20, SD  = 9.90; equivalence test trials  = 52, SD = 35.41).

### Acquisition phase

#### US expectancy ratings

**Figure 3:**
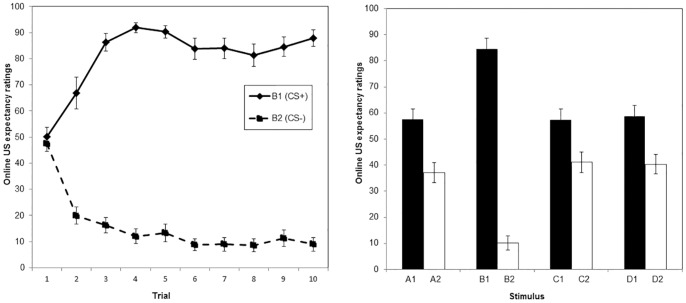
Mean expectancy ratings over de acquisition phase and generalization of acquisition test. Ratings were registered at the moment the stimulus and expectancy scale disappeared from the screen. Higher levels indicate more certainty about shock (100 =  “Certainly a shock”), lower levels indicate more certainty about absence of shock (0 =  “Certainly no shock”). The left panel represents the data from the acquisition phase, per trial, for both the CS+ (B1) and the CS- (B2). The right panel shows the data from the generalization of acquisition test of all stimuli from both categories. Each stimulus was presented once during this phase.

The left panel of Figure 3 suggests that a differential US expectancy to B1 (CS+) and B2 (CS−) emerged over the acquisition phase. This was confirmed by a mixed ANOVA, where the one between-subjects variable was condition (CS-ext group and GS-ext group) and two within-subjects variables included stimulus (B1 and B2) and trial number (1 to 10). This analysis revealed a main effect of Stimulus, *F*(1, 17)  = 1042.34, *p*<.001, partial *n^2^* = .98, and a significant interaction between Stimulus and Trial, *F*(3.17, 53.96)  = 15.85, *p*<.001, partial *n^2^* = .48. At the last acquisition trial, there was a significant difference between the two stimuli, *F*(1, 17)  = 109.18, *p*<.001, partial *n^2^* = .87. There were no differences between the two conditions (no Stimulus * Condition * Trial interaction, *F*(3.17, 53.96)  = 1.08, *p* = .37, partial *n^2^* = .05).

#### Skin conductance response

**Figure 4:**
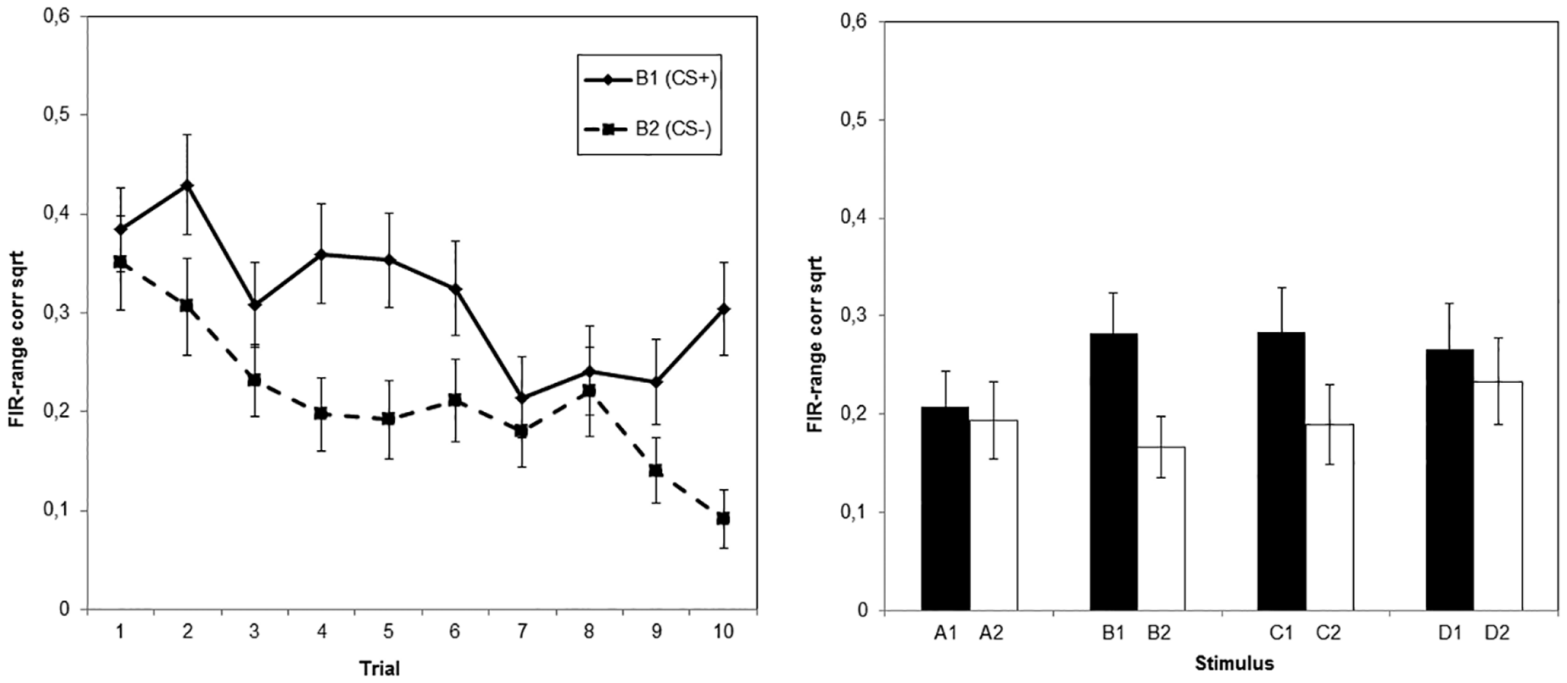
Mean skin conductance responses over the acquisition phase and generalization of acquisition test. Responses were range-corrected and square-root transformed. The left panel represents the data from the acquisition phase, per trial, for both the CS+ (B1) and the CS− (B2). The right panel shows the data from the generalization of acquisition test of all stimuli from both categories. Each stimulus was presented once during this phase.

Figure 4 represents the skin conductance responding during acquisition. The same ANOVA as the one carried out in the US expectancy data, showed no significant interaction between Stimulus and Trial, *F*(6.61, 125.56)  = 1.81, *p* = .10. However, at the last acquisition trial, there was a significant difference between the two stimuli (*F*(1, 17)  = 13.99, *p* = .002, partial *n^2^* = .45). Again, no main effect or interaction effect involving Condition was found, *F*<.42, *p*>.52.

### Generalization of acquisition test

#### US expectancy ratings

The right panel of Figure 3 shows the expectancy ratings of the first test phase. This graph suggests that CAT+ elicited higher shock expectancies than CAT−. This was analyzed by conducting a mixed ANOVA with Category (2 levels: CAT+ and CAT−) and Stimulus (4 levels: A, B, C and D) as within-subjects variables and Condition (CS-ext group and GS-ext group) as a between-subjects variable. This analysis revealed a main effect of Category, *F*(1, 27) = 77.92, *p*<.001, partial *n^2^* = .74, and an interaction between Stimulus and Category, *F*(3, 81) = 38.55, *p*<.001, partial *n^2^* = .59. There was no effect of stimulus, *F*(3, 81)  = .10, p = .96, partial *n^2^* = .004. This indicates that shock expectancies were strongly dependent on the stimulus categories created during the MTS procedure. The main effect of condition was not significant, *F*(1, 27) = .06, *p* = .81, partial *n^2^* = .002, nor was any of its interactions, *F*<1.22, *p*>.31. Planned comparisons revealed a significant difference between the mean C1 and D1 ratings versus the mean ratings of C2 and D2, *F*(1, 27) = 15.07, *p*<.001, partial *n^2^* = .38. The mean C1 rating was not different from D1 ratings and C2 did not differ from D2 ratings, *F* (1, 27)<.13, *p*>.72. This suggests that after acquisition of B1 (CS+) and B2 (CS−), shock expectancy towards C1 and D1 was elevated while lower towards C2 and D2. Stimuli A1 and A2 were also analyzed, as their relations with the B stimuli had been explicitly trained, in contrast with the relations between the B, C and D stimuli. Interestingly, in both categories the level of shock expectancies elicited by the A stimuli did not differ from the expectancies elicited by the C and D stimuli, *F*(1, 27)<1.31, *p*>.26.

It is noticeable in this test phase that generalization was not complete. The difference between the conditioned stimuli (B1 and B2) appeared to be larger than the difference between the mean ratings for CAT+ (A1, C1 and D1) and CAT− (A2, C2 and D2), *F*(1, 27)  = 82.61, *p*<.001, partial *n^2^* = .75.

#### Skin conductance response

Figure 4 suggests similar outcomes as in the shock expectancy data. This was investigated using an ANOVA with Category (2 levels: CAT+ and CAT−) and Stimulus (4 levels: A, B, C and D) as within-subjects variables and Condition (CS-ext group and GS-ext group) as a between-subjects variable. This analysis revealed a main effect of Category, *F*(1, 29) = 7.70, *p* = .01, partial *n^2^* = .21, and no interaction between Category and Stimulus, *F*(3, 87) = .93, *p* = .43, partial *n^2^* = .03. A planned comparison between C1 and D1 from CAT+ on the one hand, and C2 and D2 from CAT− on the other hand approached significance, *F*(1, 29) = 3.73, *p* = .06, partial *n^2^* = .11. Also, there was no difference between C1 and D1 skin conductance, nor between C2 and D2 skin conductance, *F*<.90, *p*>.18. Unexpectedly, there was no difference in skin conductance response between A1 and A2, *F*(1, 29) = .03, *p* = .87, partial *n^2^*<.001.

### Extinction phase

#### US expectancy ratings

**Figure 5:**
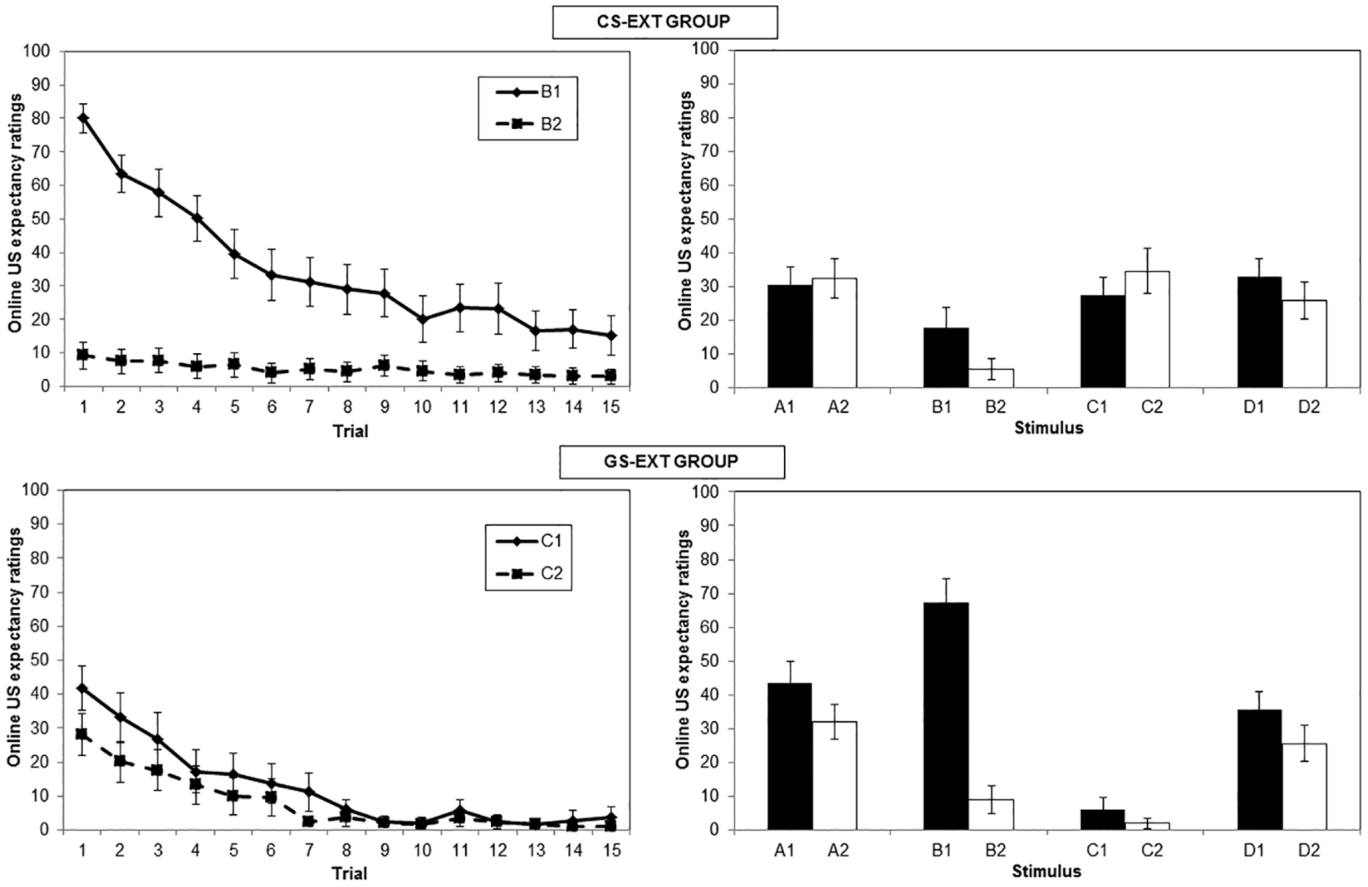
Mean expectancy ratings over the extinction phase and generalization of extinction test. Ratings were registered at the moment the stimulus and expectancy scale disappeared from the screen. Higher levels indicate more certainty about shock (100 =  “Certainly a shock”), lower levels indicate more certainty about absence of shock (0 =  “Certainly no shock”). The upper graph shows the data of CS-ext group, the lower graph the data of GS-ext group. The left panel represents the data from the extinction phase, per trial, for both B1 and B2 (group CS-ext) or C1 and C2 (group GS-ext). The right panel shows the data from the generalization of extinction test of all stimuli from both categories. Each stimulus was presented once during this phase.

Figure 5 shows the expectancy ratings during the extinction phase, for both conditions. B1 seems to elicit more US expectancy than B2 during the first trial in CS-ext group, while there is a rather small initial difference in the same direction between C1 and C2 in GS-ext group. Furthermore, in both conditions these differences seem to extinguish during this phase. This was confirmed by an ANOVA with one between-subjects variable (Condition, 2 levels: CS-ext group and GS-ext group) and two within-subjects variables (Stimulus: B1/C1 and B2/C2, and Trial: 1 to 15). This analysis revealed a significant interaction between Stimulus, Trial and Condition, *F*(3.15, 91.42) = 5.89, *p*<.001, partial *n^2^* = .31. This suggests that the difference between the stimulus from CAT+ (B1 or C1) and the stimulus from CAT- (B2 or C2) declined over the course of the extinction phase, but at a different rate in the two conditions.

At the first trial of the CS-ext group, there was a significant difference in shock expectancy between B1 and B2, *F*(1, 16) = 95.88, *p*<.001, partial *n^2^* = .86. This difference was reduced to a level below significance by the last trial, *F*(1, 15) = 3.43, *p* = .08, partial *n^2^* = .19. Moreover, there was a significant interaction between Stimulus and Trial (first vs. last), *F*(1, 32) = 57.57, *p*<.001, partial *n^2^* = .64. In the GS-ext group the difference between C1 and C2 was not significant both on the first and last trial, *F*(1, 17)>4.05, *p*>.06. Also, the Stimulus and Trial interaction was not significant, *F*(1, 32) = 2.08, *p* = .16, partial *n^2^* = .06.

#### Skin conductance response

**Figure 6:**
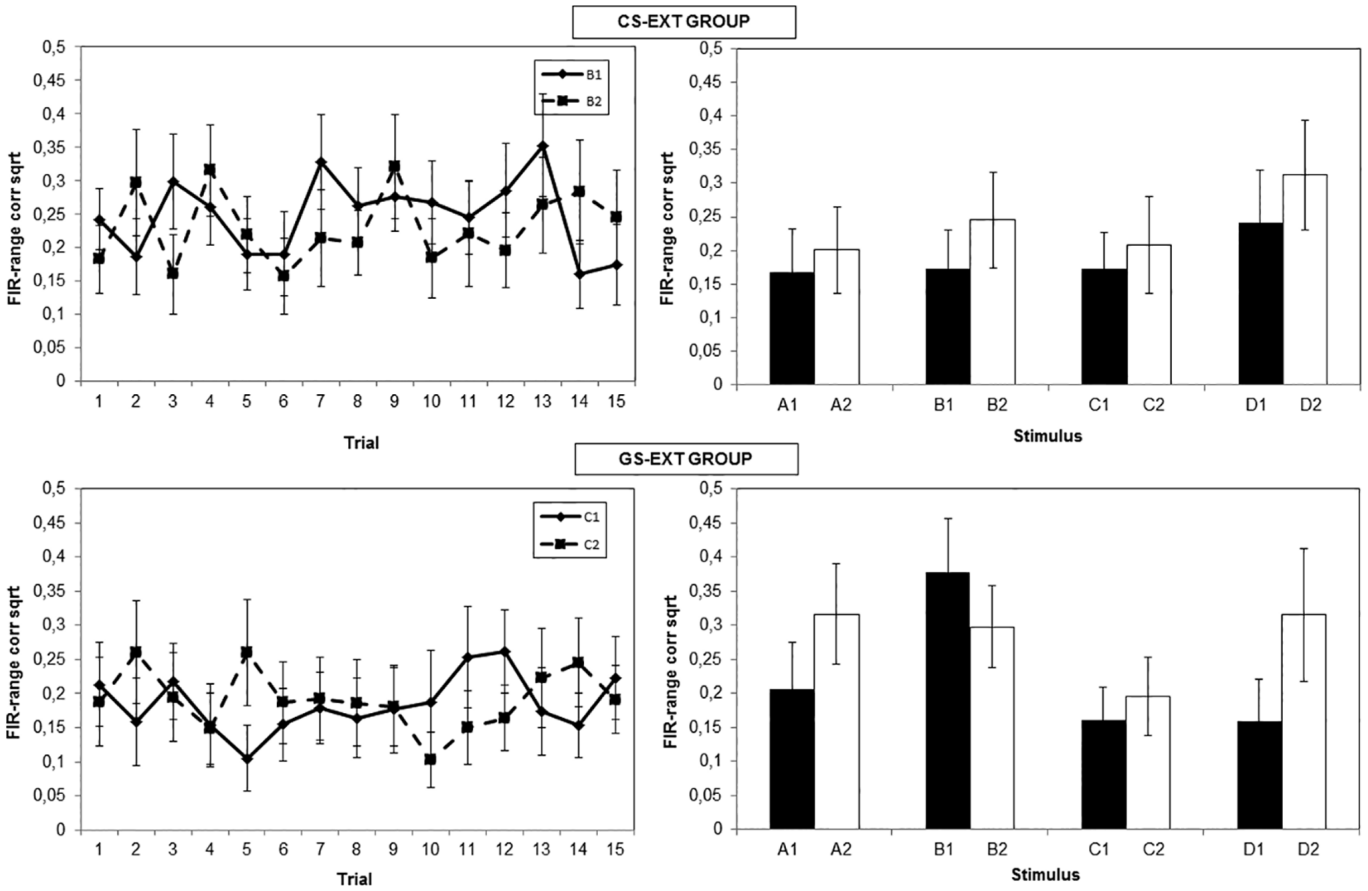
Mean skin conductance responses over the extinction phase and generalization of extinction test. Responses were range-corrected and square-root transformed. The upper graph shows the data of CS-ext group, the lower graph the data of GS-ext group. The left panel represents the data from the extinction phase, per trial, for both B1 and B2 (group CS-ext) or C1 and C2 (group GS-ext). The right panel shows the data from the generalization of extinction test phase of all stimuli from both categories. Each stimulus was presented once during this phase.

The skin conductance results in this phase appeared to be quite irregular (Figure 6). An ANOVA gave no indications of a typical extinction pattern in these data, as no main or interaction effects of Stimulus (B1/C1 and B2/C2) and Trial (1 to 15) were found, *F*<1.11, *p*>.30. Also, there was no influence of Condition (CS-ext group and GS-ext group) on the course of the extinction phase, as the Condition * Stimulus * Trial interaction was not significant, *F*(7.95, 198.75) = .50, *p* = .85, partial *n^2^* = .02.

### Generalization of extinction test

#### US expectancy ratings

Figure 5 shows the expectancy ratings towards each stimulus after extinction. Only in the GS-ext group, B1 seems to be rated considerably higher compared with the other stimuli from the first category. An ANOVA was conducted with Category (2 levels: CAT+ and CAT−) and Stimulus (4 levels: A, B, C and D) as within-subjects variables, and Condition as a between-subjects variable (CS-ext group and GS-ext group). This analysis revealed a significant interaction between Stimulus, Category and Condition, F(3, 90) = 7.53, p<.001, partial *n^2^* = .20. A series of planned comparisons did confirm some important differences between the two groups, as will be explained below.

CS-ext group: Compared with the first test phase, which took place after acquisition, the difference between B1 and B2 had declined significantly in this group, *F*(1, 25) = 49.39, *p*<.001, partial *n^2^* = .66, as expected. The difference between C1 and C2 was no longer significant in the second test, *F*(1, 30) = 1.33, *p* = .26, partial *n^2^* = .04, and was also significantly smaller compared with the first generalization test after acquisition, *F*(1, 25) = 6.69, *p* = .02, partial *n^2^* = .21. This indicates that conducting an extinction phase with the original CS+ had an effect on both the CS+ and a generalization stimulus. Likewise, the difference between D1 and D2 was no longer significant at test 2, *F*(1, 25) = 3.02, *p* = .09, partial *n^2^* = .11, in contrast with the significant difference observed in the test after acquisition.

GS-ext group: In this group, the difference between C1 and C2 was no longer significant by test 2, *F*(1, 30) = 0.59, *p* = .45, partial *n^2^* = .02, indicating that extinction of the generalized conditioned responding was complete. Interestingly, there was still a significant difference between B1 and B2 during the last test, *F*(1, 30) = 78.00, *p*<.001, partial *n^2^* = .72. There was no significant decline in the difference between B1 and B2, compared with the difference between these stimuli in the first test, *F*(1, 25) = 2.11, *p* = .16, partial *n^2^* = .08. This suggests that extinction with C1 and C2 was not effective to reduce shock expectancy towards the original CS+. There was also no evidence of generalization of extinction to the D-stimuli, as the difference between D1 and D2 was still present, *F*(1, 25) = 5.42, *p* = .03, partial *n^2^* = .18.

A final set of contrasts confirmed that the two groups differed significantly in the extent to which the differential B1/B2 responding changed from the first to the second test phase, *F*(1, 25) = 12.99, *p* = .001, partial *n^2^* = .34. The same comparison with the C-stimuli led to a non-significant result, *F*(1, 25) = .56, *p* = .46, partial *n^2^* = .02, neither was there any difference between the conditions in the evolution over tests of the difference between the D-stimuli, *F*(1, 25) = 1.50, *p* = .23, partial *n^2^* = .06.

#### Skin conductance response

Figure 6 shows the skin conductance responses towards each stimulus after extinction. The same ANOVA was conducted on the skin conductance data. The Stimulus * Category * Condition interaction was not significant, *F*(3, 75) = 1.20, *p* = .32, partial *n^2^* = .05, in contrast with the expectancy data. However, one interesting difference between the two conditions was that stimulus B1 elicited significantly less skin conductance response in CS-ext group, compared with GS-ext group, *F*(1, 25) = 8.81, *p* = .007, partial *n^2^* = .26, which again suggests that extinction with a GS was not as effective in reducing skin conductance responses towards the CS+, as extinction with the CS+ itself.

## Discussion

The present study was designed to investigate the generalization of acquisition and extinction of conditioned fear within de novo stimulus categories. First, a matching-to-sample task created two categories (CAT+ and CAT−). Next, one stimulus from CAT+ was aversively conditioned through repeated pairing with an aversive electrical stimulus, whereas one stimulus from CAT− was equally often presented but without the shock. At test, conditioned fear generalized to all CAT+ stimuli as indicated by higher shock expectancy ratings and skin conductance responses relative to CAT− stimuli. Subsequent extinction with the fear conditioned stimulus produced a decrease of shock expectancy ratings in all CAT+ stimuli. In contrast, extinction with another CAT+ stimulus had little detectable effect on the fear conditioned stimulus itself. These results are consistent with findings in the perceptual generalization area [6]–[7], [9], [27].

Fear generalization is typically studied with regard to perceptual similarity and/or associative connectivity. Stimuli that resemble the CS+ or stimuli that are associated to the CS+ elicit the conditioned fear response to a certain degree (perceptual generalization, higher order conditioning; see [28]). The current results stand out because (1) generalization spread over arbitrary stimuli with little perceptual overlap, and (2) the matching-to-sample task does not promote the formation of direct associations among category stimuli. This is because the stimuli are never experienced together but only indirectly through the central ‘sample’ stimulus. Moreover, this central stimulus is presented equally often with stimuli from its own category as with stimuli from other categories (these stimuli serve as distractor stimuli). Hence, perceptual similarity and associative connectivity mechanisms would not account for any observed differences in the generalization to the two categories. In contrast, the current results show fear generalization specifically to stimuli from the CS+ category. Fear generalization over naturalistic categories probably comprises a mixture of these three sources of generalization [10]. Hence, the current study stands out by providing evidence for generalization of conditioned responses purely based on conceptual stimulus relations.

Matching-to-sample has been used before to investigate the generalization of avoidance behaviors over de novo created categories (e.g. [29], [30]). Avoidance plays a central role in the etiology and maintenance of anxiety as it prevents extinction of acquired fears [31]. It is a central component to the behavioral dimension in the expression of fear [32]. The present study constitutes a significant extension by focusing on the two other dimensions in the expression of emotions: evaluative self-reports (indexed by US expectancy ratings) and physiological reactions (skin conductance). Together with the avoidance studies, the present study demonstrates the ability of purely conceptual categories to modulate fear generalization in humans.

The extinction of fear also generalized over stimulus categories. This was the case when the CS+ itself was extinguished, as it reduced fear of the other CAT+ stimuli. In contrast, extinguishing another CAT+ stimulus did not significantly affect fear of the CS+. These findings are consistent with perceptual generalization of fear extinction where extinguishing a different-but-similar stimulus has little effect on fear of the original CS+ [6]–[7], [9], [27]. The results surprisingly contrast with previous findings in the matching-to-sample literature. Dougher et al. [16] found strong generalization of extinction within categories and even to stimuli that were aversively conditioned. There are two important differences with the current study. First, Dougher et al. did not use statistical testing methods so some levels of fear may have gone unnoticed. Second, Dougher et al. first conditioned all stimuli from one category, to later test the effects of extinguishing only one stimulus. Pairing CAT+ stimuli with the same US (aversive electrical shock) may increase their similarity and hence strengthen the level of (extinction) generalization among them. This process is known as acquired equivalence (see [28]). Dougher et al. employed a control condition that completed a similar procedure but in the absence of the MTS phase. Here, no generalization of extinction was observed. This demonstrates that pairing a series of stimuli with the same US does not produce extinction generalization between them. However, it cannot be ruled out that this process enhances the generalization effect produced by the MTS training.

As previously discussed, Roche, Kanter, Brown, Dymond, and Fogarty [25] also investigated generalization of extinction by comparing extinction with an original CS+ to extinction with a generalization stimulus. They also reported findings incongruent with our own. In an avoidance conditioning procedure, they observed little extinction generalization from the original CS+ to a conceptually related stimulus. Extinction of the conceptually related stimulus, however, did generalize strongly to the original CS+. This set of results is entirely opposed to the current findings. A critical feature of Roche et al.'s findings is that the CS+ extinction procedure failed to reduce the avoidance response. This indicates that there was no extinction to generalize. Perhaps if the CS+ had been successfully extinguished then there would have been a notable reduction in responding to conceptually related events. On the other hand, extinguishing a conceptually related stimulus did succeed in reducing avoidance to that stimulus. The difference between these two studies draws attention to the fact that the different dimensions of emotional expression (arousal, subjective experience and action tendency) are not necessarily correlated (see [33]). That is, avoidance may remain high despite reduced conditioned arousal (e.g. [34]). In addition, the avoidance response in this study took place in a binary fashion – participants either pressed a spacebar once to prevent a US or did not. In comparison, self-reported expectancy ratings and skin conductance provide continuous measurements and thus the possibility of a more nuanced and sensitive measure. Future extinction research should include measurements of multiple dimensions of fear expression. This would enable us to construct a more complete picture of the extent to which extinction of different fear responses can generalize within stimulus categories.

One limitation to the present study is that the observed fear generalization was only partial, which may compromise a fair comparison of extinction generalization. Extinction learning requires an erroneous expectancy of the aversive US in the first place (prediction-error learning; [35]). The CS+ elicits a strong prediction of the US, yielding robust extinction learning when the US does not follow. On the other hand, a stimulus that receives only partial generalization of fear and US expectation will yield less extinction learning. Less extinction learning means less extinction generalization. As participants may have not considered the generalization stimuli to be as equally dangerous as the original CS+, it cannot be concluded that the difference in extinction generalization is more than just a difference in extinction learning. Another limitation of the procedure lies in the first generalization test phase. All stimuli were presented in extinction during this phase. This makes it difficult to interpret the generalization of extinction test, as reduced responses in this second test phase could be the result of mere extinction during the first test phase. This possibility was anticipated for by (1) using partial reinforcement during the acquisition phase, and by (2) presenting every stimulus only once during test. However, using a single test trial per stimulus does not provide ideal skin conductance measurements, as they tend to be rather variable across and within individuals. This may be one of the reasons why the skin conductance data did not reflect the expected pattern in the last part of the experiment. In addition, a test phase with one trial of each stimulus is susceptible to decreasing fear responses caused by habituation over the test phase. To control for this, two fixed stimuli (one of each category) were always presented in the first half of the test. Valverde, Luciano, and Barnes-Holmes [21] eliminated these problematic elements of the test phase by presenting all stimuli multiple times during test, in random order. Extinction was prevented by pairing all stimuli from the category that contained the CS+ with the US. Although it seems advisable to adopt these adjustments in future studies, it was decided not to so in the current study, as pairing all CAT+ members with the US would make them all CS+'s themselves. This would go against the main goal of our extinction phase, which was to compare generalization with a CS+ to generalization with a generalization stimulus.

Fear generalization between stimuli that bear physical similarity has been implicated as a central factor for the impact of a traumatic event [4]. The present study highlights the importance of conceptual knowledge as a way in which humans approach stimuli. Fear generalization processes were investigated within de novo stimulus categories. It was shown that indirect stimulus relations can radically alter responses elicited by these stimuli. Although it is too early to pass final judgment due to a few potential flaws in the design, extinction of these responses remained rather stimulus specific, unless an original conditioned stimulus was extinguished.
